# Evaluating the Benefits of Occupational Therapy in Children With Autism Spectrum Disorder Using the Autism Behavior Checklist

**DOI:** 10.7759/cureus.64012

**Published:** 2024-07-07

**Authors:** Caglar Charles D Jaicks

**Affiliations:** 1 Child and Adolescent Psychiatry, Istanbul Aydın University School of Medicine, Istanbul, TUR

**Keywords:** children, autism spectrum disorder, abc, autism, occupational therapy

## Abstract

Introduction and objective: Autism spectrum disorder (ASD) is a neurodevelopmental disorder typically manifesting before the age of three years, characterized by significant impairments in social interaction and communication, as well as restricted and repetitive patterns of interests and activities. In our study, we assessed the benefits of sensory integration therapy in children with ASD using the Autism Behavior Checklist (ABC), focusing on improvements in sensory processing, relationship-building, language skills, and social and self-care abilities.

Method: The study was conducted with 40 children aged three to nine years, diagnosed with ASD, and whose parents provided consent for their participation in therapy between December 2022 and March 2023 at the Private Adana Metro Hospital Child Psychiatry Clinic. The ages and genders of the patients were recorded. Before occupational therapy, after five sessions of occupational therapy, and after 10 sessions of occupational therapy, the ABC test was administered under the supervision of a child psychiatrist and an occupational therapist. The results were recorded, and statistical analyses were performed.

Results: In the ABC test conducted after 10 sessions, a decreasing trend was observed in sensory, relationship-building, body and object usage, language skills, social and self-care, and total scale scores compared to pre-occupational therapy test scores (p < 0.000).

Discussion and conclusion: Occupational therapy improves sensory skills, relationship-building skills, body and object usage abilities, language skills, and social and self-care skills.

## Introduction

The American Psychiatric Association defines autism spectrum disorder (ASD) in the Diagnostic and Statistical Manual of Mental Disorders, Fifth Edition (DSM-5) as a neurodevelopmental disorder characterized by persistent deficits in social communication and social interaction across multiple contexts, as well as restricted, repetitive patterns of behavior, interests, or activities. Additionally, symptoms must be present in the early developmental period and cause clinically significant impairment in social, occupational, or other important areas of current functioning. Recent reports indicate an increase in the prevalence of early-onset autism [1].

While previously categorized as a part of "Pervasive Developmental Disorders" in the Diagnostic and Statistical Manual of Mental Disorders, Fourth Edition (DSM-4), it is now classified as "Autism Spectrum Disorder" among "Neurodevelopmental Disorders" in DSM-5 and DSM-5, Text Revision (DSM-5-TR). The key differences between DSM-4 and DSM-5 include the consolidation of previously separate diagnoses (autistic disorder, Asperger’s disorder, and pervasive developmental disorder - not otherwise specified) into a single diagnosis of ASD. Additionally, DSM-5 introduced new criteria emphasizing the presence of both social communication deficits and restricted, repetitive patterns of behavior. These changes have facilitated earlier identification and intervention by recognizing a broader range of symptoms and behaviors that were previously excluded under the more rigid DSM-4 criteria [2]. There is no definitive cure for this disorder [3]. ASD is classified into three levels based on the severity of symptoms and the amount of support required: level 1 (requiring support), level 2 (requiring substantial support), and level 3 (requiring very substantial support). However, early diagnosis allows for the implementation of appropriate education to enhance adaptive skills. Various cognitive and behavioral therapies, as well as treatments for learning and speech issues, are applied in children diagnosed with autism. Some of these include sensory integration, auditory integration, music, art, drama, horseback riding therapy, and dolphin therapy [4-6]. While the effectiveness of these therapies is debatable, alternative and supportive treatments such as gluten/casein diet, vitamin/mineral supplementation, heavy metal detoxification, fungal treatment, and hyperbaric oxygen therapy can also be administered [7,8].

One of the scales developed for the assessment and screening of autism is the Autism Behavior Checklist (ABC). Developed by Krug and colleagues in 1993, commonly referred to as ABC, this scale is widely used for screening and evaluating education in autism in many countries. The ABC consists of five subscales, including sensory, relationship-building, body and object usage, language skills, and social and self-care skills, making a total of 57 items. The lowest possible score from the scale is 0 and the highest is 159. One significant advantage of the ABC is its ease of application, allowing information to be obtained from both teachers and parents. The scale has demonstrated high reliability and validity in various studies, making it a robust tool for measuring changes in autistic behaviors. Its comprehensive nature aligns well with our study objectives to evaluate improvements across multiple functional domains in children with ASD [9-14].

Occupational therapy is a therapeutic method focusing on improving activity participation skills by addressing personal characteristics, activity, and environment that affect an individual's role performance [15]. Despite various studies examining the results of sensory integration therapy in children with autism, the need for a detailed examination of the effectiveness of sensory integration therapy persists due to the limited quantity and quality of evidence-based research. Sensory integration therapy is a form of occupational therapy that aims to help individuals with sensory processing issues by providing structured sensory experiences in a controlled environment. It focuses on enhancing the brain's ability to process and respond to sensory information, which can improve functional abilities in daily activities. Sensory integration therapy is based on the principles developed by Dr. A. Jean Ayres, who emphasized the importance of sensory experiences in the development of motor skills, behavior, and learning [16]. Occupational therapists use sensory integration techniques to address sensory deficits and improve overall occupational performance in children with autism [17]. Studies such as those by Ting & Weiss and Green & Ben-Sasson have highlighted the importance of accurate assessment methods and the positive impact of sensory integration education on motor skills and daily life activities in children with autism [18,19]. Our study aims to contribute to this body of research by evaluating the benefits of occupational therapy using the ABC.

## Materials and methods

This study was designed as a prospective, observational study. The study was conducted with patients diagnosed with ASD aged three to nine years, who sought treatment at the Private Adana Metro Hospital Child Psychiatry Clinic between December 2022 and March 2023. The study employed a convenience sampling technique. The inclusion criteria were children diagnosed with ASD based on the DSM-5 criteria, aged between three and nine years, and with parental consent for participation. Children with severe comorbid medical conditions, those receiving active pharmacological treatment, those who had previously undergone occupational therapy, and those who declined to participate or did not complete the intended number of therapy sessions were excluded. Patients receiving active pharmacological treatment, those who had previously undergone occupational therapy, those who declined to participate in the study, and those who did not complete the intended number of therapy sessions were also excluded. Ethical approval was obtained from the Çukurova University Faculty of Medicine Non-invasive Clinical Research Ethics Committee (approval number: 128-58). Informed consent was obtained from the parents of all participating children. A total of 40 patient children were included in our study.

Sensory integration therapy was conducted, taking individual differences into account. It aimed to provide sensory input through proprioceptive, vestibular, and tactile senses, involving purposeful, repetitive activities requiring the child's active participation to create neural plasticity in the central nervous system. Exercises such as climbing stairs and jumping were implemented to enhance perceptual-motor skills. Occupational therapists played games with children using objects such as puzzles, pegs, and tweezers to develop fine motor skills. Floortime was provided to families to continue therapy at home.

The therapy sessions included the following: (1) proprioceptive: massage therapy, lifting heavy objects (sandbag or weight), or pushing to engage joints and muscles. Activities included walking on a sponge, climbing stairs, jumping on a trampoline, and completing obstacle course exercises. (2) Vestibular: occupational therapist-assisted exercises such as pilates, balance board, stair climbing, swinging on a swing, and balance beans. (3) Tactile: massage was applied and tactile therapy involved tactile disks, sandbags made of different fabrics, shaving, sand, soil, foam, grass, and flour. (4) Auditory: auditory therapy was conducted by playing different music in the range of 20-2000 hertz. (5) Interoceptive: massage was applied to participants. (6) Taste and smell: tasting activities included mint, vinegar, coffee, and lemon. Participants were instructed to continue exercises at home.

In our cases, to assess the benefits of occupational therapy, we chose to use the ABC scale due to its ease of application and the fact that no special training is required to administer it. This tool has been validated in Turkey for pediatric populations by Irmak et al. in 2007, ensuring its reliability and relevance in our context [20]. Although the ABC scale was originally designed to measure problematic behaviors in children over five years of age with intellectual disabilities, its utility has since been demonstrated in children with ASD across a broader age range. For our sample, which includes children aged three to nine years, no specific modifications were made to the ABC scale as existing literature supports its use in this younger age group.

Participants were evaluated by the occupational therapy expert using the ABC scale after the 5th session of sensory integration therapy, and scores were recorded. After the 10th session, they were re-evaluated using the ABC scale, and the scores obtained at the initial assessment, 5th, and 10th sessions were compared through statistical analyses.

Statistical analysis

For the analysis of all data obtained in the study, IBM SPSS version 25 software (IBM Corp., Armonk, NY) was used. Descriptive analyses of the participant group were conducted. The independent t-test or Mann-Whitney U test was employed to compare data based on the distribution characteristics. The Wilcoxon signed-rank test was used to assess the difference between two measurement results obtained from the same unit when the series were not normally distributed. Results were considered statistically significant at the p < 0.05 level.

## Results

Forty patients were included in the study. The mean age of the patients was 7.3 ± 1.4 years, with 32 (80%) patients being male (Table 1).

**Table 1:** Analyzing the descriptive characteristics of the participants.

	Frequency (n)	Percentage (%)
Gender		
Male	32	80.0
Female	8	20.0
	Mean ± SD	Med (Min-Max)
Age	7.3 ± 1.4	8 (4-9)

The primary outcome measures assessed using the ABC scale included sensory, relationship-building, body and object usage, language skills, social and self-care skills, and total scale scores. We used repeated measures ANOVA to analyze changes over time. The differences in patients' ABC measurements before occupational therapy, after five sessions, and after 10 sessions were examined and summarized for each ABC subscale separately. According to the analysis, a statistically significant decrease was observed in sensory, relationship-building, language skills, social and self-care, and total ABC scale scores (p < 0.05). However, the change observed in the body and object usage scale score was found to be not significant (p > 0.05). Upon examining the source of the identified difference with the Wilcoxon rank test, it was determined that the sensory, relationship-building, body and object usage, language skills, social and self-care, and total ABC scale scores of the patients after the 5th session showed a decreasing trend compared to the initial test findings (p < 0.05) (Table 2).

**Table 2:** Changes in ABC test results across therapy sessions. Values are presented as mean ± standard deviation. P-values were calculated with the one-way ANOVA test. The significant results are highlighted with bold characters. p1 represents the analysis of differences between the three measurements using the repeated measures test. p2 represents differences between the pre-test and values after five sessions. p3 represents differences between the pre-test and values after 10 sessions. p4 represents differences between values after five sessions and after 10 sessions. ABC: Autism Behavior Checklist.

	Pretest	After 5 sessions	After 10 sessions	p1	p2	p3	p4
	Mean ± SD	Mean ± SD	Mean ± SD				
Sensory	20.2 ± 3.4	19.3 ± 3.5	18.5 ± 3.7	<0.001	<0.001	<0.001	<0.001
Relationship building	24.8 ± 5.5	23.8 ± 5.8	22.9 ± 5.8	<0.001	<0.001	<0.001	<0.001
Body and object use	24.9 ± 5.4	22.9 ± 5.9	22.2 ± 5.9	0.060	<0.001	<0.001	0.165
Language skills	23.1 ± 4.2	21.5 ± 4.7	20.8 ± 4.9	<0.001	<0.001	<0.001	<0.001
Social and self-care	18.3 ± 4.3	17.2 ± 4.3	16.7 ± 4.5	<0.001	<0.001	<0.001	<0.001
Total	111.1 ± 17.4	104.7 ± 19.1	101.1 ± 19.7	<0.001	<0.001	<0.001	<0.001

It was observed that the sensory, relationship-building, body and object usage, language skills, social and self-care, and total ABC scale scores after the 10th session also showed a decreasing trend compared to the initial test findings (p < 0.05). Moreover, the 10th session sensory, relationship-building, language skills, social and self-care, and total ABC scale scores showed a significant decrease compared to the values after the 5th session (p < 0.05). The percentage changes in the therapies provided to the patients are detailed in Table 3. Sensory scores improved by 95.3% between the pre-test and five sessions, 91.5% between the pre-test and 10 sessions, and 96.0% between five sessions and 10 sessions. Relationship-building scores improved by 95.7% between the pre-test and five sessions, 92.2% between the pre-test and 10 sessions, and 96.3% between five sessions and 10 sessions. Body and object use scores improved by 91.8% between the pre-test and five sessions, 88.5% between the pre-test and 10 sessions, and 96.5% between five sessions and 10 sessions. Language skills scores improved by 92.7% between the pre-test and five sessions, 89.3% between the pre-test and 10 sessions, and 96.3% between five sessions and 10 sessions. Social and self-care scores improved by 94.1% between the pre-test and five sessions, 90.7% between the pre-test and 10 sessions, and 96.4% between five sessions and 10 sessions. The total ABC score improved by 93.9% between the pre-test and five sessions, 90.6% between the pre-test and 10 sessions, and 96.4% between the five sessions and 10 sessions.

**Table 3:** Percentage change in ABC scores across sessions. ABC: Autism Behavior Checklist.

	Percentage change from pretest to 5 sessions	Percentage change from pretest to 10 sessions	Percentage change from 5 sessions to 10 sessions	
				Sensory	95.3	91.5	96.0	
Relationship building	95.7	92.2	96.3	
Body and object use	91.8	88.5	96.5	
Language skills	92.7	89.3	96.3	
Social and self-care	94.1	90.7	96.4	
Total	93.9	90.6	96.4	

## Discussion

ASD is a neurodevelopmental condition characterized by various challenges. Autism manifests itself with difficulties in social interaction and communication skills, repetitive behaviors, and restricted interests or intense focus. As each individual's experience with autism can be different, the term "spectrum disorder" is used to describe it [13]. It is important to note that there is no definitive medical test to diagnose autism; its diagnosis is clinical, based on the observation of behavior and developmental history.

Our study demonstrates significant improvements in sensory processing, relationship-building, language skills, and social and self-care abilities among children who received occupational therapy. These findings underscore the value of incorporating sensory integration techniques into therapeutic programs for children with ASD. The substantial improvements observed after the initial five sessions suggest that occupational therapy has a pronounced early impact on key behavioral domains. This rapid progress is particularly notable in sensory processing and relationship-building, which are critical areas for the development of children with ASD. However, the rate of improvement tends to decrease over time, highlighting the need for sustained therapeutic interventions to maintain and build upon early gains. This finding is consistent with other studies that suggest the necessity of continuous therapy to achieve long-term benefits. Our results also emphasize the importance of early intervention. Children who began therapy at a younger age showed more significant improvements, suggesting that starting therapy early in a child’s development can lead to better outcomes.

Occupational therapy is applied to individuals with ASD to improve functional skills, increase independence in daily life activities, and enhance overall quality of life. Occupational therapy employs various strategies, techniques, and therapeutic activities to maximize the quality of life of individuals with autism and unleash their potential [14].

In individuals with ASD, the formation of incorrect responses to sensory inputs is associated with "Sensory Modulation Disorder." It has been reported that sensory integration training improves sensory modulation in children with autism [14]. Studies have discussed the benefits of sensory integration training given to children with autism and the changes it creates in them [21-24].

In our study, we based our approach on the benefits of sensory integration training and implemented a structured sensory integration therapy program. This program included activities designed to improve sensory processing, relationship-building, language skills, and social and self-care abilities, taking into account the positive changes that these therapies could bring based on evaluation parameters. The purpose was to examine the benefits of occupational therapy for autistic children, investigate its effects on gross and fine motor skills, and learn about the goals set by families for their children.

Liu et al. [25], in their research conducted in 2017, emphasized the importance of accurate assessment methods to place individuals with ASD in appropriate motor education programs. In their case study, they used four different tests to evaluate fine and gross motor skills and compared the results. Among the four assessments, they found that the Bruininks-Oseretsky Motor Proficiency Test (BOMPT-2) and Peabody Developmental Motor Scale (PDMS-2) tests performed better in measuring the child's skills. As a result, the researchers in this study emphasized the need to select tests for assessments based on the evaluation goals in studies on this subject.

In our cases, to assess the benefits of occupational therapy, we chose to use the ABC scale, which has been used for many years in autism. The reason for choosing this test was the proven Turkish validity and reliability of the test in autism [26]. While there are studies evaluating the benefits of occupational therapy in autism using various scales, the application of the ABC scale in this context appears to be limited based on our literature searches.

Karim et al. [27], in their research aiming to determine the effects of sensory integration education on motor skills and daily life activities, provided sensory integration education to 34 children with autism three times a week for six months. They reported an increase in fine and gross motor skills and a significant rise in scores on the "Goal Attainment Scale" after education.

In another study conducted in Japan, the effects of sensory integration therapy applied to children with autism were investigated. The researchers found that children with autism receiving sensory integration therapy showed improvement in non-verbal communication skills, motor coordination, sensory-motor skill abilities, and cognitive aspects compared to children with autism receiving group therapy [25]. Another study involved giving sensory integration therapy to cases, and the cases were evaluated with the "Goal Attainment Scale" when the therapy was completed. The benefits of sensory therapy are mentioned in this study, where cases are evaluated from many perspectives [28].

When we evaluated our cases using the ABC scale before occupational therapy, after five sessions, and after 10 sessions, we observed a significant decrease in the total ABC score, indicating the benefits of occupational therapy. The primary outcome measures assessed included sensory processing, relationship-building, body and object usage, language skills, and social and self-care skills. To analyze changes over time, we used repeated measures ANOVA and paired t-tests. Additionally, when the subcategories of the ABC test were individually evaluated, we found significant improvements in all skills except for body and object usage. One possible explanation for this exception could be that body and object usage skills might require more time or different types of interventions to show significant improvement compared to other skills. Specifically, sensory processing showed a significant decrease in scores, relationship-building skills also showed significant improvement, and language skills and social and self-care skills demonstrated marked progress. Although there was a decrease in scores for body and object usage, it was not statistically significant. These results are presented in a structured manner in the results section, summarizing changes in each ABC subscale separately.

Notably, we observed that the improvement after the first five sessions was more significant compared to the development after 10 sessions. This result may suggest that the initial impact of occupational therapy is substantial, but the rate of improvement might decrease over time.

One of the primary strengths of this study is the use of the ABC scale, a widely recognized and validated tool for assessing changes in behavior in children with ASD. The study's design, which included repeated measures at three distinct time points (pre-therapy, after five sessions, and after 10 sessions), allowed for a detailed analysis of the progression and impact of occupational therapy on various behavioral domains. Additionally, the relatively large sample size (40 participants) and the inclusion of a diverse age range (three to nine years) enhance the generalizability of the findings.

Despite these strengths, the study has several limitations. First, the lack of a control group means that we cannot definitively attribute the observed improvements to occupational therapy alone; other factors such as natural developmental progress or external interventions might have influenced the outcomes. Second, the study's duration was relatively short (three months), which limits our understanding of the long-term effects of occupational therapy. Third, the reliance on parent-reported measures may introduce bias, as parents might overestimate or underestimate their child's progress. Finally, while the ABC scale is comprehensive, it may not capture all nuances of behavioral change, and additional measures could provide a more complete picture.

## Conclusions

Based on the findings of our study, we conclude that occupational therapy significantly improves sensory skills, relationship-building abilities, body and object usage, language skills, and social and self-care skills in children with ASD. The empirical evidence from our study indicates that these benefits are particularly pronounced in the early stages of therapy, though the rate of improvement tends to decrease over time. This suggests that while occupational therapy can effectively enhance the quality of life and daily life participation for children with ASD, sustained and possibly varied interventions may be necessary to maintain and build upon these gains.

Our study highlights the potential of occupational therapy as a valuable intervention for children with ASD. However, it is important to acknowledge the limitations of our study, including the relatively short duration, lack of a control group, and reliance on parent-reported measures, which may introduce bias. Future research should aim to examine the long-term effects of occupational therapy and explore its impact on children with different profiles of ASD in more detail. Additionally, investigating the integration of other therapeutic modalities could provide a more comprehensive approach to treatment and further enhance outcomes for children with ASD.
